# Solitary Wave in One-dimensional Buckyball System at Nanoscale

**DOI:** 10.1038/srep21052

**Published:** 2016-02-19

**Authors:** Jun Xu, Bowen Zheng, Yilun Liu

**Affiliations:** 1 Department of Automotive Engineering, School of Transportation Science and Engineering, Beihang University, Beijing, China, 100191; 2 Advanced Vehicle Research Center, Beihang University, Beijing, China, 100191; 3 Beijing Key Laboratory for High-efficient Power Transmission and System Control of New Energy Resource Vehicle, Beihang University, Beijing 100191, China; 4 State Key Laboratory for Strength and Vibration of Mechanical Structures, School of Aerospace Engineering, Xi’an Jiaotong University, Xi’an, China, 710049

## Abstract

We have studied the stress wave propagation in one-dimensional (1-D) nanoscopic buckyball (C_60_) system by molecular dynamics (MD) simulation and quantitative modeling. Simulation results have shown that solitary waves are generated and propagating in the buckyball system through impacting one buckyball at one end of the buckyball chain. We have found the solitary wave behaviors are closely dependent on the initial temperature and impacting speed of the buckyball chain. There are almost no dispersion and dissipation of the solitary waves (stationary solitary wave) for relatively low temperature and high impacting speed. While for relatively high temperature and low impacting speed the profile of the solitary waves is highly distorted and dissipated after propagating several tens of buckyballs. A phase diagram is proposed to describe the effect of the temperature and impacting speed on the solitary wave behaviors in buckyball system. In order to quantitatively describe the wave behavior in buckyball system, a simple nonlinear-spring model is established, which can describe the MD simulation results at low temperature very well. The results presented in this work may lay a solid step towards the further understanding and manipulation of stress wave propagation and impact energy mitigation at nanoscale.

Wave propagation in one-dimensional granular chains at macroscale has been an attractive topic in recent 30 years. The existence of solitary waves in granular medium was first theoretically predicted by Nesterenko in 1983^1^, and was experimentally observed by Lazaridi and Nesterenko in 1985^2^. The interaction between adjacent elastic granules is governed by Hertz law. Such a system can exhibit linear, weakly nonlinear and strongly nonlinear regimes depending on the precompression applied to the system. Because of the strongly nonlinear contact force at zero precompression, some unique responses have been found in the macroscopic granular chains, such as the “sonic vacuum” (zero sound velocity), the highly nonlinear solitary wave and the strongly nonlinear dependence of wave speed on wave amplitude^3^, which is qualitatively different from the well-known weakly nonlinear solitary wave first observed by John Scott Russell^4^, where it is the solution of the famous Korteweg-de Vries (KdV) equation. Comprehensive studies related to this topic were conducted in various governing aspects, for example, the effect of physical properties such as the material and the shape of granules^5^,^6^,^7^,^8^,^9^,^10^, the effect of granule arrangements^11^,^12^,^13^,^14^, the attenuation feature of the wave propagation along the chains^15^,^16^,^17^,^18^,^19^ and the energy absorption ability of the granular system^20^,^21^.

Buckminsterfullerene (or buckyball), is an elastic sphere molecule with the formula C_60_, C_180_, C_240_, C_320_, etc. Xu *et al*.^22^,^23^,^24^ first studied 1-D buckyball chains at nanoscale using MD simulation and showed the buckyball system an excellent candidate for the impacting energy mitigation and force attenuation purpose. The buckyball chain and the macroscopic granular chain share a large amount of similarities, e.g. the same features of discrete nature and one-dimensionality. Indeed, the fullerene family has usually been described by continuum elastic spherical model^25^. However, the interaction between two buckyballs is contributed by the van der Waals interactions among all carbon atoms on the two buckyballs which can have force interaction in both compression and tension, which is completely different from the Hertz law in macroscopic granular system without precompression. The strongly nonlinear contact force plays the major role in the solitary wave behaviors of macroscopic granular system. So, it is very interesting to probe into the wave behavior in 1-D buckyball chains and identify whether the nanoscopic discrete system with van der Waals interaction supports solitary wave propagation. On the other hand, at nanoscale the thermal fluctuation may be comparable to the solitary wave amplitude. Therefore, it is also important to study the temperature effect on the solitary wave behaviors of 1-D buckyball chains. The understanding of the stress wave propagation behavior at nanoscale discrete system is beneficial to design and tune the dynamical mechanical properties of the nanoscale metamaterials for the current emerging applications of shock wave protection, impact energy mitigation, tunable acoustic devices, etc.

## Results

In this work, MD simulations are conducted first at various initial system temperatures and impacting speeds to study the stress wave propagation in 1-D buckyball chains. The effects of the initial system temperature and impacting speed on the solitary wave behaviors are explored. Then, taking account of the nonlinear force-displacement relation of inter-buckyballs, a simple nonlinear-spring model is established which can quantitatively describe the MD simulation results at low temperature very well. It is found the nonlinear inter-buckyballs interaction can be approximately described by a power law, but the power index is larger than the value of the macroscopic elastic spheres governed by the Hertz law which shows a stronger nonlinearity of the inter-buckyballs interaction. Based on the nonlinear-spring model, the relation between the solitary wave speed and the wave amplitude is derived which agrees well with MD simulation results.

**Figure 1:**
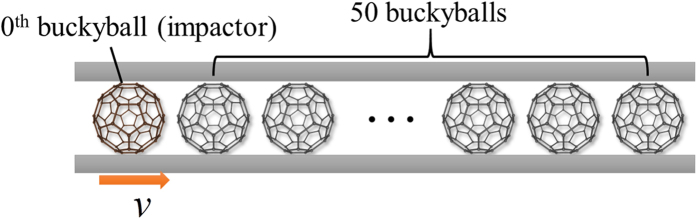
Illustration of one-dimensional buckyball chain. 0th buckyball serves as the wave generator with an initial speed *v*. Other 50 buckyballs are at the equilibrium positions. An ideal frictionless rigid wall tightly holds the chain to guarantee the one dimension wave propagation.

The nanoscopic system investigated in this work is introduced as follows (see Fig. 1). The 1-D buckyball chain is composed of 51 C_60_ buckyballs with space of *d*_0_ = 10.05 Å (the equilibrium distance of two C_60_ molecules^26^) for adjacent buckyballs. The 0^th^ buckyball, with an initial speed *v*, serves as the impactor to generate a wave propagating along the chain. The impactor is also a buckyball (C_60_) so as to prevent the occurrence of shock wave, which may result from a long contact time^3^. The whole system is tightly held by an ideal frictionless rigid lateral wall in order to maintain a 1-D wave propagation along the axis of the chain. Due to the short-range property of inter-buckyballs interaction, only the adjacent buckyballs have van der Waals interaction. And the adjacent buckyballs space equals to the equilibrium distance, so the initial force on each buckyball caused by other molecules is zero, which is similar to the zero precompression cases of the macroscopic counterpart in previous research^1^,^2^,^27^.

To investigate the wave propagation behaviors in this system, MD simulations are carried out based on LAMMPS (large-scale atomic/molecular massively parallel simulator) platform^28^. The full atomistic description of the buckyballs is applied in our MD simulations. The carbon-carbon interaction of intra-buckyball is described by the adaptive intermolecular reactive empirical bond order (AIREBO) potential which has been widely used to simulate the buckyball, carbon nanotube and graphene system^29^,^30^,^31^, while the carbon-carbon interaction of inter-buckyballs is accounted for by van der Waals interactions, described by a pairwise Lennard-Jones (L-J) potential term 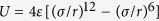. Here the inter-buckyballs van der Waals interactions plays the major role in the solitary wave propagation of the buckyball chains and according to Girifalco’s work^26^, the parameters in the L-J potential for C_60_-C_60_ interactions are chosen as *σ* = 3.4656 Å and *ε* = 0.0658 Kcal/mol slightly different from L-J potential parameters for interlayer graphene interactions. The time integration step is set as 1 fs. Firstly, the system is running for equilibrium for 30 ps in the NVT ensemble (the canonical ensemble) so as to set an initial temperature for the system before the impacting. Later, as shown in Fig. 1, an initial speed *v* is added to the 0^th^ buckyball to generate the solitary wave in the buckyball chains and the buckyball system is simulated with NVE ensemble (the micro-canonical ensemble) for next 10 ps to study the solitary wave propagation behaviors in the buckyball chains.

**Figure 2:**
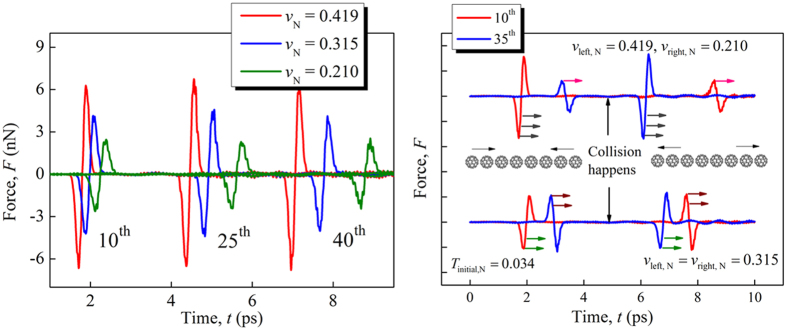
MD simulation results of the stress wave propagation in 1-D buckyball chain at initial temperature *T*_initial, N_ = 0.034. (**a**) Force histories form the 10^th^, 25^th^ and 40^th^ buckyballs for three impact speeds, i.e. *v*_N_ = 0.210, 0.315 and 0.419 (the stress waves are generated at left end). Negative force value represents right resultant force. (**b**) Force histories from the 10^th^ and 35^th^ buckyballs (the stress waves are generated at both ends). Two pairs of impact speeds are investigated, i.e. *v*_N, left_ = *v*_N, right_ = 0.315; *v*_N, left_ = 0.419, *v*_N, right_ = 0.210. The vertical scale is 4 nN. Individual waves are represented by arrows of distinguishing colors.

Firstly, we have simulated the 1-D buckyball chains at low initial temperature (*T*_initial_ = 10 K, which is further normalized as *T*_initial,N_ = *T*_initial_/*T*_0_ = 0.034, where *T*_0_ = 293 K) for different impacting speeds *v* = 500 m/s, 750 m/s and 1000 m/s, which are normalized as *v*_N_ = 0.210, 0.315 and 0.419 using one-tenth of the wave speed 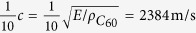 respectively. Here, Young’s modulus *E* = 3.10 GPa and 
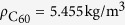
. Resultant force histories are extracted from 10th, 25th and 40th buckyballs to represent the wave signals, as shown in Fig. 2(a). At the low initial system temperature the profile of the stress wave is perfected kept and almost no dissipation is observed for the wave propagation of the 50 C_60_ buckyballs in our MD simulations. The wave speed and amplitude increase with the impacting speed, which is similar to the solitary wave in macroscopic granular chains governed by Hertz law. However, as shown in Fig. 2(a), the stress wave in the buckyball chains is composed of one symmetrical positive and negative stress pulses, and the width of the wave decreases with increasing the impacting speed, different from the solitary waves in macroscopic granular chains without precompression (the wave width is constant). We attribute these differences of the solitary wave behaviors are caused by the different interactions in buckyball chains (van der Waals interaction) and macroscopic granular chains (Hertz law).

It is also necessary to see how two waves behave after interacting with each other. According to Drazen and Johnson^32^, solitary waves remain unchanged after the collision with another solitary wave. To this end, impacts are generated at both ends by giving 0^th^ and 50^th^ buckyballs initial speeds of opposite directions (*v*_left_ and *v*_right_ respectively). Simulation results show that in both cases whether the stress waves are identical or different, both of the profile and amplitude of the two interacting waves remain unaltered after collision (see Fig. 2(b)), which further confirms the solitary wave propagation in 1-D buckyball chains.

**Figure 3:**
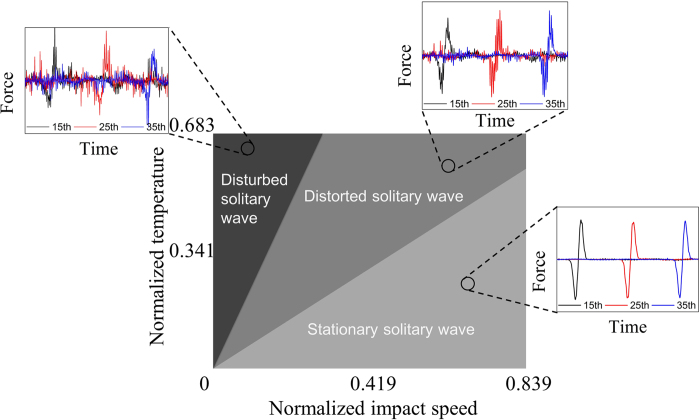
The phase diagram of the solitary waves in 1-D buckyball chains for different initial system temperatures and impacting speeds.

For the C_60_ buckyball chains, the thermal fluctuation may be comparable to the solitary wave amplitude. So the temperature effect plays an important role in the solitary wave behaviors of the buckyball chains. While for the macroscopic granular chains the thermal fluctuation is trivial, so that the temperature effect on the solitary wave behaviors has not been studied. The solitary wave behaviors in 1-D buckyball chains for different initial system temperatures and impacting speeds have been studied via MD simulations. Note that the system temperature may increase after the solitary wave propagation due to the dissipation. Generally, the solitary wave behaviors in 1-D buckyball chains can be divided into three regions, as shown in Fig. 3. For relatively low initial system temperature (*T*_initial, N_ < 0.341) and high impacting speed (*v*_N_ > 0.419) (the lower right region in Fig. 3), the profile of the solitary waves are perfectly kept and almost no dissipation is observed for the propagation 50 buckyballs in our simulations. We define this region as stationary solitary wave region. In contract, for relatively high initial system temperature (*T*_initial, N_ > 0.341) and low impacting speed (*v*_N_ < 0.419) (the upper left region of Fig. 3), the solitary wave is highly distorted by the random disturbance of the temperature effect, so that the profiles of the solitary waves in different buckyballs are completely different. Besides, the solitary waves are dissipated after propagation of several tens of buckyballs. We call this region as the disturbed solitary wave region. In the middle region, the solitary wave is also distorted and dissipated. But, the profile of the solitary wave in each buckyball is similar. We call this region the distorted solitary wave region. Note that the boundaries between the adjacent regions are not exactly clear and Fig. 3 is just a qualitative description of the three regions. From the perspective of molecular movement these phenomena can be explained. The thermal vibration of the molecules increases with temperature, leading to increasingly drastic uncertain disturbance to the wave propagation. The temperature effect is essentially different from the viscosity effect in macroscopic granular chains, though both of the two effects cause the dissipation of the solitary waves. The quantitative description of the temperature effect on the solitary wave behaviors in nanoscale granular systems should be systematically studied in future works.

**Figure 4:**
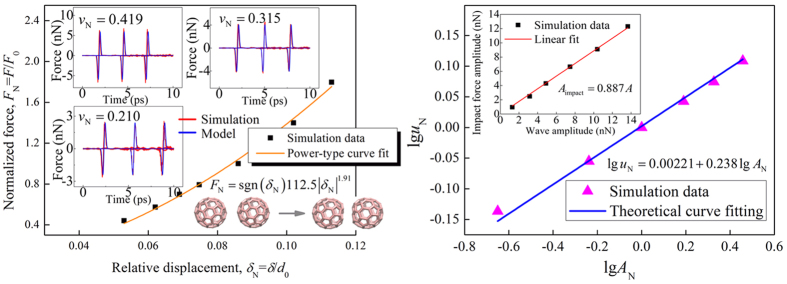
The nonlinear-spring model of 1-D buckyball chains. (**a**) the inter-buckyballs force-displacement relation. Here, the force *F* and displacement *δ* are normalized as *F*_N_= *F*/*F*_0_ and *δ*_N_ = *δ*/*d*_0_, respectively, where *F*_0_ = 3.24 nN corresponding to the inter-buckyball force at displacement *δ* = 0.86 Å and *d*_0_ = 10.05 Å is the equilibrium spacing of two buckyballs. The insets are the comparison between the nonlinear-spring predictions and MD simulation results at low temperature (*T*_initial, N_ = 0.034 K) for different impacting speeds, i.e. *v*_N_ = 0.210, 0.315 and 0.419. (**b**) The relation between the wave speed and wave amplitude. The symbols are obtained from MD simulations at *T*_initial, N_ = 0.034 for impact speeds, i.e. 0.105, 0.210, 0.315, 0.419, 0.524, 0.629. The inset is the relation between the solitary wave amplitude and the maximum force at the impactor.

It is critical to establish a quantitative physical model to describe the stress wave behaviors in 1-D buckyball chains. For simplicity and referring to the nonlinear-spring model used in macroscale granular chains, a nonlinear-spring model is proposed to describe the stress wave behaviors in 1-D buckyball chains. Based on the nonlinear force-displacement relation obtained from the collision of two C_60_ buckyballs, the nonlinear spring can be depicted as 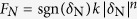. Here, the force *F* and displacement *δ* are normalized as *F*_*N*_ = *F*/*F*_0_ and *δ*_N_ = *δ*/*d*_0_, respectively, where *F*_0_ = 3.24 nN corresponding to the inter-buckyballs force at displacement *δ* = 0.86 Å. Note that the compression displacement is set as positive value to consist with the definition in macroscopic granular chains. As the van der Waals interaction, there is a tensile force for tensile displacement which is different from the Hertz law in macroscopic granular chains. By fitting to the MD simulation results, the two parameters are *k* = 112.5 and *n* = 1.91, as shown in Fig. 4(a). The index *n* = 1.91 here is larger than the value for the interaction between two elastic solid spheres (*n* = 1.5)^3^, suggesting a stronger nonlinearity of inter-buckyballs interactions than its macroscopic counterpart. Then, the equation of motion for the *i*th buckyball can be written as


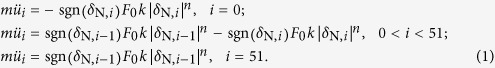


where *δ*_N,*i*_ = (*u*_*i*+1_ − *u*_*i*_)/*d*_0_ and *u*_*i*_ is the displacement of the *i*th buckyball; *m* = 1.2 × 10^−24^ kg is the mass of a C_60_ buckyball.

Based on the nonlinear-spring model, the stress waves in 1-D buckyball chains are obtained by numerically solving Eq. (1). A comparison is made between the solutions of equation (1) and the MD simulation results at low temperature (*T*_initial, N_ = 0.034) for different impacting speeds, i.e. *v*_N_ = 0.210, 0.315 and 0.419. As shown in Fig. 4(a), this nonlinear-spring model agrees well with the MD simulation results. This remarkable consistency reveals that nonlinear van der Waals interactions between adjacent buckyballs play the major role in the solitary wave behaviors of 1-D buckyball chains at low temperature. The nonlinear-spring model accurately captures the nonlinear inter-buckyballs force-displacement relation, so that it can describe the solitary wave behaviors in 1-D buckyball chain at low temperature very well. The temperature effect is not considered in current nonlinear-spring model which will be included in our future works.

Next, the relation between the solitary wave speed and wave amplitude is discussed. Based on the nonlinear-spring model used in the macroscopic granular chains, the dependence of solitary wave speed on amplitude is *u* ∝ *A*^(*n*−1)/2*n*^, where *u* is wave speed and *A* is wave amplitude^3^. By substituting the power index of the inter-buckyballs force-displacement relation *n* = 1.91 into *u* ∝ *A*^(*n*−1)/2*n*^, the relation between the normalized solitary wave speed and amplitude can be well depicted as *u*_N_ = 1.005 × *A*_N_^0.238^, as shown in Fig. 4(b), where (*n* − 1)/2*n* = 0.238, *u*_N_ and *A*_N_ are normalized wave speed and amplitude respectively, i.e. *u*_N_ = *u*/*u*_0_, *A*_N_ = *A*/*A*_0_. The wave speed and wave amplitude is plotted in logarithmic coordinates for the clear view of the power relation. Here, *u*_0_ = 5181 m/s and *A*_0_ = 4.29 nN are the wave speed and amplitude corresponding to the impact speed *v*_N_ = 0.315. Furthermore, a linear relation between the solitary wave amplitude and the maximum force at the impactor is found, i.e. *A*_impact_ = 0.887*A*, as shown in the inset of Fig. 4(b). As the impacting energy is distributed among several granules during the solitary wave propagation, the wave amplitude is smaller than the impact force amplitude. Therefore, the solitary wave speed can be further represented by the impactor force amplitude as *u*_N_ = 1.034 × (*A*_impact_/*A*_0_)^0.238^.

In this work, the stress wave behaviors in 1-D buckyball system have been investigated by MD simulation and quantitative modeling. The principal differences between the nanoscale buckyball system and the macroscale granular system lie in the different interaction laws (van der Waals interaction for buckyball system and Hertz law for macroscale granular system) and temperature effect. Due to the discrete nature and strong nonlinear interaction of buckyball chains, the solitary wave propagation is found in the 1-D buckyball chains. Different from the solitary wave in macroscale granular system, the solitary waves in buckyball chains are composed of one symmetrical positive and negative force pulses which is caused by the van der Waals interaction in buckyball system. The temperature effect plays an important role in the solitary wave behaviors of the buckyball system. A phase diagram is proposed in which the solitary wave behaviors are divided into three regions, i.e. the stationary solitary wave region (relatively low initial system temperature and high impacting speed), the disturbed solitary wave region (relatively high initial system temperature and low impacting speed) and the distorted solitary wave region. A nonlinear-spring model is established by accurately describing the van der Waals force-displacement relation of inter-buckyballs with a nonlinear spring, which can describe the MD simulation results at low temperature very well. This work may provide a powerful tool to investigate a series of related problem and open a new area to study the solitary wave propagation at nanoscale.

## Additional Information

**How to cite this article**: Xu, J. *et al*. Solitary Wave in One-dimensional Buckyball System at Nanoscale. *Sci. Rep*. **6**, 21052; doi: 10.1038/srep21052 (2016).
